# Crystal structure of (1,3-di­methyl­thio­urea-κ*S*)tris­(tri­phenyl­phosphane-κ*P*)silver(I) acetate

**DOI:** 10.1107/S1600536814019047

**Published:** 2014-08-30

**Authors:** Yupa Wattanakanjana, Arunpatcha Nimthong, Chanokphat Darasuriyong

**Affiliations:** a Department of Chemistry, Faculty of Science, Prince of Songkla University, Hat Yai, Songkhla 90112, Thailand; b Department of Chemistry, Youngstown State University, 1 University Plaza, 44555 Youngstown, OH, USA

**Keywords:** crystal structure, 1,3-di­methyl­thio­urea, silver complex

## Abstract

In the mononuclear title salt, [Ag(C_3_H_8_N_2_S)(C_18_H_15_P)_3_](CH_3_COO), the Ag^I^ ion exhibits a distorted tetra­hedral coordination sphere defined by three P atoms from three tri­phenyl­phosphane ligands and one S atom from a 1,3-di­­methyl­thio­urea ligand. In the crystal, the acetate anion is linked with the complex cation *via* duplex N—H⋯O hydrogen bonds [graph-set motif *R* ^2^_2_(8)].

## Related literature   

For studies of silver(I) complexes with tertiary phosphane and sulfur-donor ligands as co-ligands, see: McFarlane *et al.* (1998 ▶); Lobana *et al.* (2008 ▶); Pakawatchai *et al.* (2012 ▶). For potential applications of silver(I) complexes, see: Isab *et al.* (2010 ▶); Ferrari *et al.* (2007 ▶). The observed bond lengths distribution is in good agreement with related structures, such as [Ag_2_Cl_2_(μ-*S*-H*L*)_2_(PPh_3_)_2_] (H*L* = 2-benzoyl­pyridine thio­semicarbazone; Lobana *et al.*, 2008 ▶) and [Ag(C_5_H_12_N_2_S)(C_18_H_15_P)_3_](CH_3_COO)·CH_3_OH (Wattanakanjana *et al.*, 2014 ▶). For graph-set analysis, see: Etter *et al.* (1990 ▶).
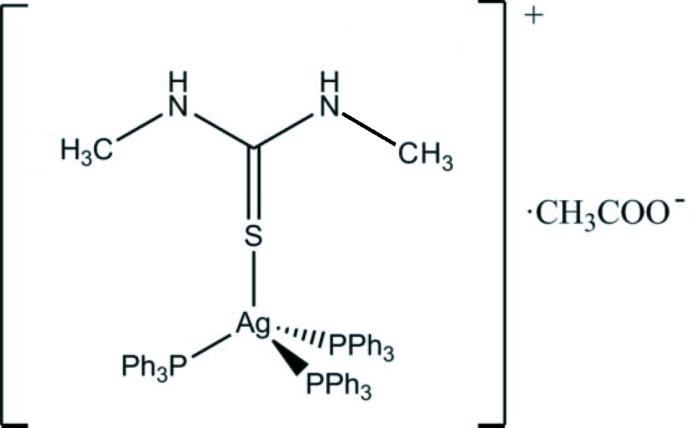


## Experimental   

### Crystal data  

[Ag(C_3_H_8_N_2_S)(C_18_H_15_P)_3_]C_2_H_3_O_2_
*M* *_r_* = 1057.89
Monoclinic, 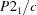

*a* = 15.780 (4) Å
*b* = 15.427 (4) Å
*c* = 21.649 (6) Å
β = 102.262 (5)°
*V* = 5150 (2) Å^3^
*Z* = 4
Mo *K*α radiation
μ = 0.57 mm^−1^
*T* = 100 K
0.15 × 0.09 × 0.07 mm

### Data collection  

Bruker APEXII CCD diffractometer
Absorption correction: multi-scan (*SADABS*; Bruker, 2013 ▶) *T*_min_ = 0.628, *T*_max_ = 0.746
26460 measured reflections
11781 independent reflections
7575 reflections with *I* > 2σ(*I*)
*R*_int_ = 0.074

### Refinement  

*R*[*F* ^2^ > 2σ(*F* ^2^)] = 0.053
*wR*(*F* ^2^) = 0.106
*S* = 1.02
11781 reflections
616 parameters
H-atom parameters constrained
Δρ_max_ = 0.80 e Å^−3^
Δρ_min_ = −0.64 e Å^−3^

### 

Data collection: *APEX2* (Bruker, 2013 ▶); cell refinement: *SAINT* (Bruker, 2013 ▶); data reduction: *SAINT*; program(s) used to solve structure: *SHELXS97* (Sheldrick, 2008 ▶); program(s) used to refine structure: *SHELXL2013* (Sheldrick, 2008 ▶) and *SHELXLE* (Hübschle *et al.*, 2011 ▶); molecular graphics: *Mercury* (Macrae *et al.*, 2008 ▶); software used to prepare material for publication: *SHELXL97* (Sheldrick, 2008 ▶) and *publCIF* (Westrip, 2010 ▶).

## Supplementary Material

Crystal structure: contains datablock(s) I, New_Global_Publ_Block. DOI: 10.1107/S1600536814019047/wm5052sup1.cif

Structure factors: contains datablock(s) I. DOI: 10.1107/S1600536814019047/wm5052Isup2.hkl

**Fig. 1.**
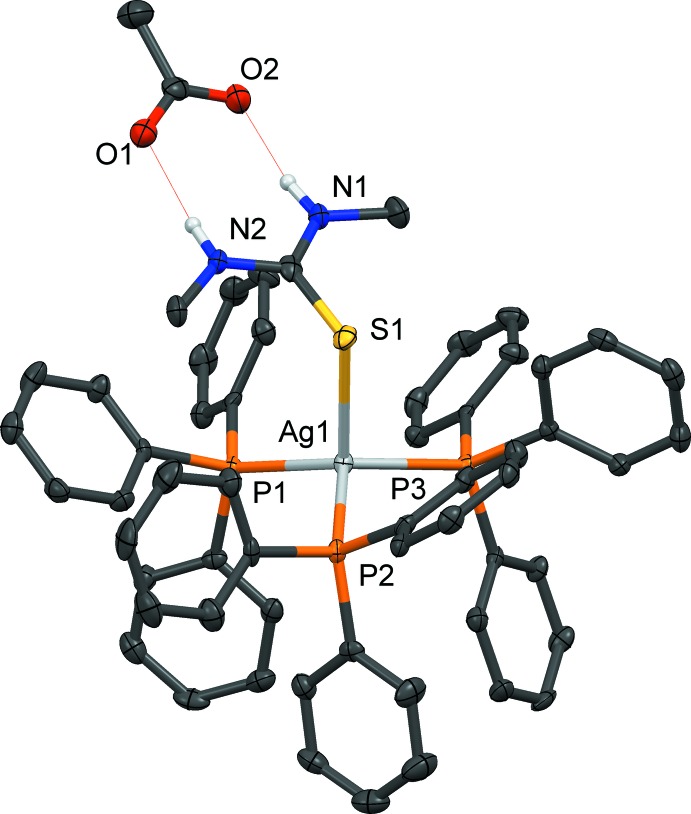
The molecular entities of the title compound with displacement ellipsoids drawn at the 50% probability level. N—H···O hydrogen bonds are shown as red lines.

. DOI: 10.1107/S1600536814019047/wm5052fig1.tif
The mol­ecular entities of the title compound with displacement ellipsoids drawn at the 50% probability level. N—H⋯O hydrogen bonds are shown as red lines.

**Fig. 2.**
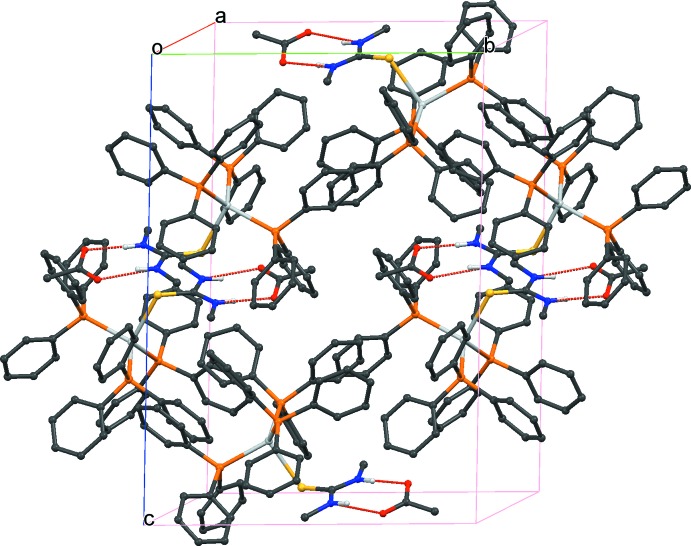
Packing plot of the molecular components of the title compound. N—H···O hydrogen bonds are shown as red lines.

. DOI: 10.1107/S1600536814019047/wm5052fig2.tif
Packing plot of the mol­ecular components of the title compound. N—H⋯O hydrogen bonds are shown as red lines.

CCDC reference: 1020689

Additional supporting information:  crystallographic information; 3D view; checkCIF report

## Figures and Tables

**Table 1:** Hydrogen-bond geometry (Å, °)

*D*—H⋯*A*	*D*—H	H⋯*A*	*D*⋯*A*	*D*—H⋯*A*
N1—H1⋯O2	0.88	1.89	2.762 (4)	170
N2—H2⋯O1	0.88	1.91	2.773 (4)	166

## supplementary crystallographic information

### S1. Synthesis and crystallisation

Tri­phenyl­phosphane, PPh_3_, (0.31 g) was dissolved in 30 ml of acetone at 338 K and then silver acetate, AgOAc, (0.10 g) was added. The mixture was stirred for 2 hr and then *N*,*N*'-di­methyl­thio­urea, dmtu, (0.07 g) was added and the new reaction mixture was heated under reflux for 4 hr during which the precipitate gradually disappeared. The resulting clear solution was filtered and left to evaporate at room temperature. The crystalline complex, which deposited upon standing for several days, was filtered off and dried in *vacuo*.

### S2. Refinement

Reflections (002), (100), (111), (110), (102) and (011) were affected by the beam stop and were omitted from the refinement. H atoms bonded to C and N atoms were included in calculated positions and were refined with a riding model using distances of 0.95 Å (aryl H), and *U*_iso_(H) = 1.2*U*_eq_(C); 0.98 Å (CH_3_) and *U*_iso_(H) = 1.5*U*_eq_(C); 0.88 Å (NH), and *U*_iso_(H) = 1.2*U*_eq_(N).

### Crystal data

**Table:**

[Ag(C_3_H_8_N_2_S)(C_18_H_15_P)_3_]C_2_H_3_O_2_	*F*(000) = 2192
*M**_r_* = 1057.89	*D*_x_ = 1.365 Mg m^−^^3^
Monoclinic, *P*2_1_/*c*	Mo *K*α radiation, λ = 0.71073 Å
*a* = 15.780 (4) Å	Cell parameters from 2479 reflections
*b* = 15.427 (4) Å	θ = 2.2–22.0°
*c* = 21.649 (6) Å	µ = 0.57 mm^−^^1^
β = 102.262 (5)°	*T* = 100 K
*V* = 5150 (2) Å^3^	Rod, colourless
*Z* = 4	0.15 × 0.09 × 0.07 mm

### Data collection

**Table:**

Bruker APEXII CCD diffractometer	11781 independent reflections
Radiation source: fine focus sealed tube	7575 reflections with *I* > 2σ(*I*)
Graphite monochromator	*R*_int_ = 0.074
ω and phi scans	θ_max_ = 27.5°, θ_min_ = 2.3°
Absorption correction: multi-scan (*SADABS*; Bruker, 2013)	*h* = −20→17
*T*_min_ = 0.628, *T*_max_ = 0.746	*k* = −20→11
26460 measured reflections	*l* = −28→27

### Refinement

**Table:**

Refinement on *F*^2^	Primary atom site location: structure-invariant direct methods
Least-squares matrix: full	Secondary atom site location: difference Fourier map
*R*[*F*^2^ > 2σ(*F*^2^)] = 0.053	Hydrogen site location: inferred from neighbouring sites
*wR*(*F*^2^) = 0.106	H-atom parameters constrained
*S* = 1.02	*w* = 1/[σ^2^(*F*_o_^2^) + (0.0281*P*)^2^] where *P* = (*F*_o_^2^ + 2*F*_c_^2^)/3
11781 reflections	(Δ/σ)_max_ = 0.001
616 parameters	Δρ_max_ = 0.80 e Å^−^^3^
0 restraints	Δρ_min_ = −0.64 e Å^−^^3^

### Special details

**Table:**

Geometry. All e.s.d.'s (except the e.s.d. in the dihedral angle between two l.s. planes) are estimated using the full covariance matrix. The cell e.s.d.'s are taken into account individually in the estimation of e.s.d.'s in distances, angles and torsion angles; correlations between e.s.d.'s in cell parameters are only used when they are defined by crystal symmetry. An approximate (isotropic) treatment of cell e.s.d.'s is used for estimating e.s.d.'s involving l.s. planes.

### Fractional atomic coordinates and isotropic or equivalent isotropic displacement parameters (Å^2^)

**Table:**

	*x*	*y*	*z*	*U*_iso_*/*U*_eq_	
C1	0.3031 (2)	0.5069 (3)	0.94747 (18)	0.0208 (9)	
C2	0.4133 (3)	0.5809 (3)	0.9015 (2)	0.0332 (11)	
H2A	0.4041	0.5466	0.8626	0.050*	
H2B	0.4619	0.5566	0.9325	0.050*	
H2C	0.4264	0.6410	0.8922	0.050*	
C3	0.1888 (2)	0.4489 (3)	0.9976 (2)	0.0248 (10)	
H3A	0.1775	0.4004	0.9677	0.037*	
H3B	0.1338	0.4705	1.0057	0.037*	
H3C	0.2258	0.4291	1.0373	0.037*	
C4	0.1342 (2)	0.4805 (2)	0.75494 (18)	0.0172 (9)	
C5	0.1944 (3)	0.5404 (3)	0.78514 (19)	0.0218 (9)	
H5	0.2357	0.5239	0.8219	0.026*	
C6	0.1950 (3)	0.6247 (3)	0.7622 (2)	0.0278 (10)	
H6	0.2357	0.6656	0.7838	0.033*	
C7	0.1367 (3)	0.6488 (3)	0.7084 (2)	0.0286 (11)	
H7	0.1375	0.7062	0.6927	0.034*	
C8	0.0771 (3)	0.5900 (3)	0.6772 (2)	0.0282 (10)	
H8	0.0370	0.6064	0.6398	0.034*	
C9	0.0762 (3)	0.5060 (3)	0.70093 (19)	0.0234 (10)	
H9	0.0347	0.4655	0.6795	0.028*	
C10	0.0642 (2)	0.3820 (2)	0.84535 (17)	0.0139 (8)	
C11	0.0312 (2)	0.4598 (3)	0.86199 (18)	0.0216 (9)	
H11	0.0453	0.5124	0.8436	0.026*	
C12	−0.0221 (3)	0.4614 (3)	0.9051 (2)	0.0270 (10)	
H12	−0.0436	0.5153	0.9165	0.032*	
C13	−0.0442 (3)	0.3862 (3)	0.93169 (19)	0.0253 (10)	
H13	−0.0817	0.3878	0.9607	0.030*	
C14	−0.0110 (2)	0.3072 (3)	0.91574 (19)	0.0230 (9)	
H14	−0.0254	0.2548	0.9342	0.028*	
C15	0.0422 (2)	0.3058 (2)	0.87344 (19)	0.0211 (9)	
H15	0.0647	0.2519	0.8629	0.025*	
C16	0.0758 (2)	0.3035 (2)	0.72943 (17)	0.0173 (9)	
C17	−0.0135 (2)	0.3085 (3)	0.71026 (18)	0.0214 (9)	
H17	−0.0445	0.3495	0.7296	0.026*	
C18	−0.0583 (3)	0.2549 (3)	0.66341 (19)	0.0258 (10)	
H18	−0.1196	0.2592	0.6507	0.031*	
C19	−0.0136 (3)	0.1946 (3)	0.63501 (19)	0.0268 (10)	
H19	−0.0438	0.1583	0.6021	0.032*	
C20	0.0756 (3)	0.1878 (3)	0.65507 (19)	0.0249 (9)	
H20	0.1064	0.1454	0.6368	0.030*	
C21	0.1197 (3)	0.2422 (2)	0.70136 (18)	0.0198 (9)	
H21	0.1810	0.2377	0.7142	0.024*	
C22	0.2110 (2)	0.1580 (2)	0.95183 (18)	0.0174 (9)	
C23	0.2183 (3)	0.2192 (3)	0.99953 (19)	0.0256 (10)	
H23	0.2574	0.2663	1.0010	0.031*	
C24	0.1689 (3)	0.2122 (3)	1.0452 (2)	0.0363 (12)	
H24	0.1751	0.2540	1.0781	0.044*	
C25	0.1110 (3)	0.1451 (3)	1.0433 (2)	0.0363 (12)	
H25	0.0768	0.1405	1.0743	0.044*	
C26	0.1035 (3)	0.0843 (3)	0.9954 (2)	0.0388 (12)	
H26	0.0637	0.0377	0.9938	0.047*	
C27	0.1534 (3)	0.0903 (3)	0.9494 (2)	0.0298 (11)	
H27	0.1477	0.0481	0.9167	0.036*	
C28	0.2627 (2)	0.0767 (2)	0.84717 (17)	0.0165 (9)	
C29	0.1843 (3)	0.0723 (3)	0.80319 (19)	0.0226 (9)	
H29	0.1436	0.1181	0.8012	0.027*	
C30	0.1646 (3)	0.0031 (3)	0.7625 (2)	0.0275 (10)	
H30	0.1109	0.0015	0.7326	0.033*	
C31	0.2231 (3)	−0.0637 (3)	0.7652 (2)	0.0344 (12)	
H31	0.2095	−0.1119	0.7376	0.041*	
C32	0.3015 (3)	−0.0607 (3)	0.8080 (2)	0.0378 (12)	
H32	0.3419	−0.1066	0.8094	0.045*	
C33	0.3214 (3)	0.0089 (3)	0.8489 (2)	0.0294 (11)	
H33	0.3755	0.0105	0.8782	0.035*	
C34	0.3878 (2)	0.1533 (2)	0.95073 (18)	0.0159 (8)	
C35	0.3990 (2)	0.0966 (2)	1.00142 (18)	0.0173 (9)	
H35	0.3515	0.0624	1.0077	0.021*	
C36	0.4778 (3)	0.0889 (2)	1.04271 (19)	0.0224 (9)	
H36	0.4843	0.0495	1.0771	0.027*	
C37	0.5479 (3)	0.1383 (3)	1.03452 (19)	0.0229 (9)	
H37	0.6021	0.1337	1.0636	0.027*	
C38	0.5384 (3)	0.1941 (3)	0.98399 (19)	0.0246 (10)	
H38	0.5864	0.2275	0.9776	0.030*	
C39	0.4592 (2)	0.2016 (2)	0.94246 (18)	0.0190 (9)	
H39	0.4533	0.2403	0.9077	0.023*	
C40	0.3827 (2)	0.2476 (2)	0.70976 (17)	0.0135 (8)	
C41	0.3577 (2)	0.1651 (2)	0.72423 (19)	0.0196 (9)	
H41	0.3427	0.1546	0.7638	0.023*	
C42	0.3547 (3)	0.0980 (3)	0.6812 (2)	0.0235 (10)	
H42	0.3373	0.0417	0.6913	0.028*	
C43	0.3766 (3)	0.1125 (3)	0.6241 (2)	0.0241 (10)	
H43	0.3751	0.0660	0.5951	0.029*	
C44	0.4007 (2)	0.1943 (3)	0.60837 (18)	0.0221 (9)	
H44	0.4151	0.2044	0.5685	0.026*	
C45	0.4037 (2)	0.2616 (2)	0.65126 (17)	0.0181 (9)	
H45	0.4204	0.3179	0.6406	0.022*	
C46	0.5064 (2)	0.3314 (2)	0.80672 (17)	0.0143 (8)	
C47	0.5333 (2)	0.3744 (2)	0.86439 (18)	0.0184 (9)	
H47	0.4920	0.4046	0.8824	0.022*	
C48	0.6194 (3)	0.3733 (2)	0.89543 (19)	0.0213 (9)	
H48	0.6369	0.4033	0.9344	0.026*	
C49	0.6805 (3)	0.3290 (3)	0.87027 (19)	0.0249 (10)	
H49	0.7395	0.3280	0.8919	0.030*	
C50	0.6548 (3)	0.2863 (3)	0.8134 (2)	0.0266 (10)	
H50	0.6965	0.2562	0.7957	0.032*	
C51	0.5687 (2)	0.2867 (2)	0.78180 (19)	0.0198 (9)	
H51	0.5519	0.2565	0.7429	0.024*	
C52	0.3793 (2)	0.4305 (2)	0.71993 (17)	0.0140 (8)	
C53	0.4453 (3)	0.4913 (2)	0.72276 (19)	0.0225 (9)	
H53	0.4986	0.4846	0.7525	0.027*	
C54	0.4326 (3)	0.5617 (3)	0.6817 (2)	0.0287 (11)	
H54	0.4773	0.6037	0.6842	0.034*	
C55	0.3567 (3)	0.5714 (3)	0.6378 (2)	0.0267 (10)	
H55	0.3494	0.6191	0.6095	0.032*	
C56	0.2910 (3)	0.5114 (3)	0.63488 (19)	0.0233 (10)	
H56	0.2381	0.5179	0.6047	0.028*	
C57	0.3025 (3)	0.4423 (2)	0.67590 (18)	0.0195 (9)	
H57	0.2567	0.4017	0.6740	0.023*	
C58	0.2149 (3)	0.7483 (3)	0.9683 (2)	0.0267 (10)	
C59	0.1905 (3)	0.8391 (3)	0.9867 (2)	0.0456 (14)	
H59A	0.1960	0.8420	1.0326	0.068*	
H59B	0.1305	0.8518	0.9656	0.068*	
H59C	0.2293	0.8818	0.9738	0.068*	
N1	0.3354 (2)	0.5787 (2)	0.92701 (16)	0.0228 (8)	
H1	0.3078	0.6278	0.9291	0.027*	
N2	0.2324 (2)	0.5181 (2)	0.97095 (15)	0.0206 (8)	
H2	0.2107	0.5707	0.9703	0.025*	
O1	0.17981 (18)	0.68572 (18)	0.99000 (14)	0.0293 (7)	
O2	0.26766 (18)	0.74290 (17)	0.93254 (15)	0.0338 (8)	
S1	0.35100 (6)	0.40759 (7)	0.94540 (5)	0.0203 (2)	
Ag1	0.28404 (2)	0.31848 (2)	0.84130 (2)	0.01465 (8)	
P1	0.13742 (6)	0.37329 (6)	0.79102 (5)	0.0148 (2)	
P2	0.28410 (6)	0.17238 (6)	0.89731 (5)	0.0148 (2)	
P3	0.39152 (6)	0.33287 (6)	0.76932 (5)	0.0134 (2)	

### Atomic displacement parameters (Å^2^)

**Table:**

	*U*^11^	*U*^22^	*U*^33^	*U*^12^	*U*^13^	*U*^23^
C1	0.015 (2)	0.031 (3)	0.015 (2)	−0.0006 (18)	−0.0013 (17)	−0.0036 (17)
C2	0.027 (3)	0.035 (3)	0.042 (3)	−0.009 (2)	0.018 (2)	−0.002 (2)
C3	0.017 (2)	0.032 (3)	0.028 (2)	0.0002 (19)	0.0106 (19)	0.0012 (19)
C4	0.021 (2)	0.013 (2)	0.020 (2)	0.0042 (17)	0.0114 (18)	−0.0023 (16)
C5	0.020 (2)	0.024 (2)	0.021 (2)	−0.0009 (18)	0.0046 (18)	0.0037 (18)
C6	0.031 (3)	0.022 (2)	0.030 (3)	−0.012 (2)	0.007 (2)	0.0001 (19)
C7	0.029 (3)	0.020 (2)	0.039 (3)	0.0021 (19)	0.012 (2)	0.009 (2)
C8	0.025 (2)	0.029 (3)	0.029 (3)	0.008 (2)	0.001 (2)	0.010 (2)
C9	0.017 (2)	0.024 (2)	0.029 (3)	0.0029 (18)	0.0028 (18)	−0.0006 (19)
C10	0.0069 (18)	0.019 (2)	0.015 (2)	0.0000 (16)	0.0014 (15)	0.0007 (16)
C11	0.023 (2)	0.022 (2)	0.021 (2)	0.0046 (18)	0.0084 (18)	0.0038 (18)
C12	0.034 (3)	0.026 (3)	0.026 (2)	0.013 (2)	0.016 (2)	0.0048 (19)
C13	0.022 (2)	0.033 (3)	0.023 (2)	0.004 (2)	0.0097 (19)	0.0022 (19)
C14	0.024 (2)	0.024 (2)	0.024 (2)	−0.0085 (19)	0.0106 (18)	−0.0016 (18)
C15	0.022 (2)	0.015 (2)	0.027 (2)	0.0023 (18)	0.0071 (18)	−0.0020 (17)
C16	0.022 (2)	0.015 (2)	0.014 (2)	−0.0007 (17)	0.0037 (16)	−0.0001 (16)
C17	0.017 (2)	0.025 (2)	0.021 (2)	0.0038 (18)	0.0029 (17)	−0.0003 (18)
C18	0.019 (2)	0.034 (3)	0.023 (2)	−0.005 (2)	0.0021 (19)	0.0062 (19)
C19	0.039 (3)	0.024 (3)	0.015 (2)	−0.011 (2)	−0.0001 (19)	−0.0026 (18)
C20	0.031 (2)	0.018 (2)	0.026 (2)	0.001 (2)	0.0070 (19)	−0.0037 (19)
C21	0.021 (2)	0.017 (2)	0.023 (2)	0.0014 (17)	0.0097 (18)	0.0047 (17)
C22	0.0105 (19)	0.022 (2)	0.022 (2)	0.0047 (16)	0.0071 (16)	0.0090 (17)
C23	0.026 (2)	0.028 (3)	0.026 (2)	−0.0024 (19)	0.014 (2)	0.0010 (19)
C24	0.042 (3)	0.039 (3)	0.034 (3)	0.007 (2)	0.021 (2)	−0.001 (2)
C25	0.032 (3)	0.049 (3)	0.037 (3)	0.008 (2)	0.027 (2)	0.011 (2)
C26	0.030 (3)	0.048 (3)	0.044 (3)	−0.010 (2)	0.018 (2)	0.009 (2)
C27	0.026 (3)	0.032 (3)	0.033 (3)	−0.002 (2)	0.010 (2)	0.007 (2)
C28	0.022 (2)	0.013 (2)	0.015 (2)	−0.0021 (16)	0.0058 (17)	0.0035 (16)
C29	0.017 (2)	0.024 (2)	0.026 (2)	−0.0013 (18)	0.0031 (18)	0.0019 (18)
C30	0.026 (2)	0.027 (3)	0.027 (3)	−0.010 (2)	0.000 (2)	−0.0022 (19)
C31	0.046 (3)	0.025 (3)	0.031 (3)	−0.007 (2)	0.005 (2)	−0.008 (2)
C32	0.043 (3)	0.028 (3)	0.039 (3)	0.013 (2)	0.001 (2)	−0.008 (2)
C33	0.028 (2)	0.027 (3)	0.028 (3)	0.013 (2)	−0.005 (2)	−0.0010 (19)
C34	0.017 (2)	0.014 (2)	0.016 (2)	0.0057 (16)	0.0024 (16)	0.0000 (16)
C35	0.015 (2)	0.018 (2)	0.020 (2)	0.0019 (17)	0.0074 (17)	−0.0012 (17)
C36	0.025 (2)	0.022 (2)	0.020 (2)	0.0068 (19)	0.0032 (18)	0.0050 (17)
C37	0.014 (2)	0.029 (3)	0.024 (2)	0.0055 (18)	0.0012 (18)	−0.0014 (18)
C38	0.017 (2)	0.028 (3)	0.029 (2)	−0.0015 (19)	0.0063 (18)	0.0013 (19)
C39	0.021 (2)	0.021 (2)	0.018 (2)	0.0046 (17)	0.0097 (17)	0.0029 (16)
C40	0.0114 (19)	0.019 (2)	0.0108 (19)	0.0037 (16)	0.0030 (15)	−0.0006 (15)
C41	0.018 (2)	0.021 (2)	0.022 (2)	−0.0001 (17)	0.0081 (17)	−0.0004 (17)
C42	0.023 (2)	0.015 (2)	0.035 (3)	0.0020 (18)	0.011 (2)	−0.0027 (19)
C43	0.023 (2)	0.021 (2)	0.026 (2)	0.0083 (18)	0.0019 (19)	−0.0124 (19)
C44	0.027 (2)	0.024 (3)	0.015 (2)	0.0060 (19)	0.0063 (17)	0.0012 (17)
C45	0.021 (2)	0.021 (2)	0.014 (2)	0.0030 (17)	0.0071 (17)	0.0015 (17)
C46	0.0123 (19)	0.014 (2)	0.016 (2)	0.0003 (16)	0.0020 (15)	0.0034 (16)
C47	0.016 (2)	0.019 (2)	0.020 (2)	0.0007 (17)	0.0058 (17)	0.0019 (17)
C48	0.024 (2)	0.019 (2)	0.018 (2)	−0.0077 (18)	−0.0014 (18)	0.0023 (17)
C49	0.015 (2)	0.028 (3)	0.030 (2)	−0.0014 (19)	0.0031 (18)	0.012 (2)
C50	0.016 (2)	0.033 (3)	0.033 (3)	0.0031 (19)	0.0091 (19)	0.001 (2)
C51	0.018 (2)	0.019 (2)	0.023 (2)	−0.0018 (17)	0.0068 (18)	−0.0047 (17)
C52	0.017 (2)	0.012 (2)	0.016 (2)	0.0026 (16)	0.0097 (16)	−0.0023 (15)
C53	0.022 (2)	0.016 (2)	0.025 (2)	−0.0012 (18)	−0.0043 (18)	0.0006 (17)
C54	0.033 (3)	0.018 (2)	0.034 (3)	−0.0036 (19)	0.005 (2)	0.0023 (19)
C55	0.040 (3)	0.016 (2)	0.024 (2)	0.003 (2)	0.007 (2)	0.0083 (18)
C56	0.022 (2)	0.028 (3)	0.020 (2)	0.0097 (19)	0.0035 (18)	0.0038 (18)
C57	0.021 (2)	0.017 (2)	0.021 (2)	−0.0014 (17)	0.0066 (18)	0.0022 (17)
C58	0.020 (2)	0.025 (3)	0.030 (3)	−0.001 (2)	−0.005 (2)	−0.007 (2)
C59	0.043 (3)	0.031 (3)	0.062 (4)	−0.001 (2)	0.008 (3)	−0.014 (2)
N1	0.0183 (19)	0.022 (2)	0.031 (2)	−0.0017 (15)	0.0126 (16)	−0.0025 (16)
N2	0.0160 (18)	0.0199 (19)	0.028 (2)	0.0004 (15)	0.0102 (15)	−0.0040 (15)
O1	0.0270 (16)	0.0262 (17)	0.0367 (18)	0.0018 (14)	0.0113 (14)	−0.0009 (14)
O2	0.0232 (17)	0.0270 (18)	0.053 (2)	−0.0014 (14)	0.0129 (16)	−0.0016 (15)
S1	0.0204 (5)	0.0248 (6)	0.0158 (5)	0.0024 (4)	0.0040 (4)	−0.0058 (4)
Ag1	0.01253 (14)	0.01663 (16)	0.01532 (15)	0.00167 (13)	0.00415 (11)	0.00066 (13)
P1	0.0119 (5)	0.0173 (6)	0.0155 (5)	0.0021 (4)	0.0034 (4)	−0.0004 (4)
P2	0.0115 (5)	0.0176 (6)	0.0161 (5)	0.0016 (4)	0.0047 (4)	0.0027 (4)
P3	0.0133 (5)	0.0142 (6)	0.0137 (5)	0.0003 (4)	0.0048 (4)	−0.0001 (4)

### Geometric parameters (Å, º)

**Table:**

C1—N2	1.332 (5)	C31—C32	1.379 (6)
C1—N1	1.334 (5)	C31—H31	0.9500
C1—S1	1.714 (4)	C32—C33	1.384 (6)
C2—N1	1.450 (5)	C32—H32	0.9500
C2—H2A	0.9800	C33—H33	0.9500
C2—H2B	0.9800	C34—C35	1.385 (5)
C2—H2C	0.9800	C34—C39	1.394 (5)
C3—N2	1.454 (5)	C34—P2	1.816 (4)
C3—H3A	0.9800	C35—C36	1.373 (5)
C3—H3B	0.9800	C35—H35	0.9500
C3—H3C	0.9800	C36—C37	1.385 (5)
C4—C9	1.381 (5)	C36—H36	0.9500
C4—C5	1.386 (5)	C37—C38	1.375 (5)
C4—P1	1.825 (4)	C37—H37	0.9500
C5—C6	1.393 (5)	C38—C39	1.380 (5)
C5—H5	0.9500	C38—H38	0.9500
C6—C7	1.372 (6)	C39—H39	0.9500
C6—H6	0.9500	C40—C41	1.388 (5)
C7—C8	1.376 (6)	C40—C45	1.393 (5)
C7—H7	0.9500	C40—P3	1.826 (4)
C8—C9	1.395 (5)	C41—C42	1.387 (5)
C8—H8	0.9500	C41—H41	0.9500
C9—H9	0.9500	C42—C43	1.371 (5)
C10—C11	1.386 (5)	C42—H42	0.9500
C10—C15	1.401 (5)	C43—C44	1.380 (5)
C10—P1	1.820 (4)	C43—H43	0.9500
C11—C12	1.383 (5)	C44—C45	1.387 (5)
C11—H11	0.9500	C44—H44	0.9500
C12—C13	1.373 (5)	C45—H45	0.9500
C12—H12	0.9500	C46—C47	1.398 (5)
C13—C14	1.397 (5)	C46—C51	1.399 (5)
C13—H13	0.9500	C46—P3	1.822 (4)
C14—C15	1.368 (5)	C47—C48	1.382 (5)
C14—H14	0.9500	C47—H47	0.9500
C15—H15	0.9500	C48—C49	1.384 (5)
C16—C17	1.385 (5)	C48—H48	0.9500
C16—C21	1.386 (5)	C49—C50	1.379 (6)
C16—P1	1.825 (4)	C49—H49	0.9500
C17—C18	1.382 (5)	C50—C51	1.384 (5)
C17—H17	0.9500	C50—H50	0.9500
C18—C19	1.388 (5)	C51—H51	0.9500
C18—H18	0.9500	C52—C57	1.386 (5)
C19—C20	1.385 (6)	C52—C53	1.393 (5)
C19—H19	0.9500	C52—P3	1.833 (4)
C20—C21	1.378 (5)	C53—C54	1.390 (5)
C20—H20	0.9500	C53—H53	0.9500
C21—H21	0.9500	C54—C55	1.370 (6)
C22—C27	1.378 (5)	C54—H54	0.9500
C22—C23	1.386 (5)	C55—C56	1.381 (5)
C22—P2	1.831 (4)	C55—H55	0.9500
C23—C24	1.387 (6)	C56—C57	1.375 (5)
C23—H23	0.9500	C56—H56	0.9500
C24—C25	1.375 (6)	C57—H57	0.9500
C24—H24	0.9500	C58—O1	1.252 (5)
C25—C26	1.384 (6)	C58—O2	1.255 (5)
C25—H25	0.9500	C58—C59	1.529 (6)
C26—C27	1.399 (6)	C59—H59A	0.9800
C26—H26	0.9500	C59—H59B	0.9800
C27—H27	0.9500	C59—H59C	0.9800
C28—C29	1.392 (5)	N1—H1	0.8800
C28—C33	1.393 (5)	N2—H2	0.8800
C28—P2	1.821 (4)	S1—Ag1	2.6595 (11)
C29—C30	1.377 (5)	Ag1—P1	2.4866 (11)
C29—H29	0.9500	Ag1—P3	2.5450 (11)
C30—C31	1.376 (6)	Ag1—P2	2.5592 (11)
C30—H30	0.9500		
			
N2—C1—N1	115.5 (4)	C36—C35—H35	119.5
N2—C1—S1	122.4 (3)	C34—C35—H35	119.5
N1—C1—S1	122.1 (3)	C35—C36—C37	120.4 (4)
N1—C2—H2A	109.5	C35—C36—H36	119.8
N1—C2—H2B	109.5	C37—C36—H36	119.8
H2A—C2—H2B	109.5	C38—C37—C36	119.4 (4)
N1—C2—H2C	109.5	C38—C37—H37	120.3
H2A—C2—H2C	109.5	C36—C37—H37	120.3
H2B—C2—H2C	109.5	C37—C38—C39	120.1 (4)
N2—C3—H3A	109.5	C37—C38—H38	119.9
N2—C3—H3B	109.5	C39—C38—H38	119.9
H3A—C3—H3B	109.5	C38—C39—C34	121.1 (4)
N2—C3—H3C	109.5	C38—C39—H39	119.5
H3A—C3—H3C	109.5	C34—C39—H39	119.5
H3B—C3—H3C	109.5	C41—C40—C45	118.8 (3)
C9—C4—C5	118.3 (4)	C41—C40—P3	118.9 (3)
C9—C4—P1	125.0 (3)	C45—C40—P3	122.2 (3)
C5—C4—P1	116.7 (3)	C42—C41—C40	120.3 (4)
C4—C5—C6	120.7 (4)	C42—C41—H41	119.9
C4—C5—H5	119.6	C40—C41—H41	119.9
C6—C5—H5	119.6	C43—C42—C41	120.3 (4)
C7—C6—C5	120.0 (4)	C43—C42—H42	119.9
C7—C6—H6	120.0	C41—C42—H42	119.9
C5—C6—H6	120.0	C42—C43—C44	120.5 (4)
C6—C7—C8	120.2 (4)	C42—C43—H43	119.8
C6—C7—H7	119.9	C44—C43—H43	119.8
C8—C7—H7	119.9	C43—C44—C45	119.4 (4)
C7—C8—C9	119.4 (4)	C43—C44—H44	120.3
C7—C8—H8	120.3	C45—C44—H44	120.3
C9—C8—H8	120.3	C44—C45—C40	120.8 (4)
C4—C9—C8	121.3 (4)	C44—C45—H45	119.6
C4—C9—H9	119.4	C40—C45—H45	119.6
C8—C9—H9	119.4	C47—C46—C51	118.2 (3)
C11—C10—C15	118.2 (4)	C47—C46—P3	118.4 (3)
C11—C10—P1	123.8 (3)	C51—C46—P3	123.3 (3)
C15—C10—P1	117.9 (3)	C48—C47—C46	120.6 (4)
C12—C11—C10	120.4 (4)	C48—C47—H47	119.7
C12—C11—H11	119.8	C46—C47—H47	119.7
C10—C11—H11	119.8	C47—C48—C49	120.6 (4)
C13—C12—C11	120.9 (4)	C47—C48—H48	119.7
C13—C12—H12	119.6	C49—C48—H48	119.7
C11—C12—H12	119.6	C50—C49—C48	119.3 (4)
C12—C13—C14	119.4 (4)	C50—C49—H49	120.3
C12—C13—H13	120.3	C48—C49—H49	120.3
C14—C13—H13	120.3	C49—C50—C51	120.7 (4)
C15—C14—C13	119.6 (4)	C49—C50—H50	119.7
C15—C14—H14	120.2	C51—C50—H50	119.7
C13—C14—H14	120.2	C50—C51—C46	120.5 (4)
C14—C15—C10	121.4 (4)	C50—C51—H51	119.7
C14—C15—H15	119.3	C46—C51—H51	119.7
C10—C15—H15	119.3	C57—C52—C53	118.6 (3)
C17—C16—C21	118.6 (4)	C57—C52—P3	118.5 (3)
C17—C16—P1	122.4 (3)	C53—C52—P3	122.8 (3)
C21—C16—P1	119.0 (3)	C54—C53—C52	119.5 (4)
C18—C17—C16	121.1 (4)	C54—C53—H53	120.2
C18—C17—H17	119.5	C52—C53—H53	120.2
C16—C17—H17	119.5	C55—C54—C53	121.0 (4)
C17—C18—C19	119.8 (4)	C55—C54—H54	119.5
C17—C18—H18	120.1	C53—C54—H54	119.5
C19—C18—H18	120.1	C54—C55—C56	119.7 (4)
C20—C19—C18	119.3 (4)	C54—C55—H55	120.1
C20—C19—H19	120.3	C56—C55—H55	120.1
C18—C19—H19	120.3	C57—C56—C55	119.7 (4)
C21—C20—C19	120.3 (4)	C57—C56—H56	120.1
C21—C20—H20	119.8	C55—C56—H56	120.1
C19—C20—H20	119.8	C56—C57—C52	121.5 (4)
C20—C21—C16	120.8 (4)	C56—C57—H57	119.3
C20—C21—H21	119.6	C52—C57—H57	119.3
C16—C21—H21	119.6	O1—C58—O2	125.8 (4)
C27—C22—C23	119.7 (4)	O1—C58—C59	116.9 (4)
C27—C22—P2	124.6 (3)	O2—C58—C59	117.3 (4)
C23—C22—P2	115.7 (3)	C58—C59—H59A	109.5
C22—C23—C24	120.6 (4)	C58—C59—H59B	109.5
C22—C23—H23	119.7	H59A—C59—H59B	109.5
C24—C23—H23	119.7	C58—C59—H59C	109.5
C25—C24—C23	120.4 (4)	H59A—C59—H59C	109.5
C25—C24—H24	119.8	H59B—C59—H59C	109.5
C23—C24—H24	119.8	C1—N1—C2	124.1 (3)
C24—C25—C26	119.0 (4)	C1—N1—H1	117.9
C24—C25—H25	120.5	C2—N1—H1	117.9
C26—C25—H25	120.5	C1—N2—C3	124.2 (3)
C25—C26—C27	121.1 (4)	C1—N2—H2	117.9
C25—C26—H26	119.4	C3—N2—H2	117.9
C27—C26—H26	119.4	C1—S1—Ag1	112.73 (13)
C22—C27—C26	119.3 (4)	P1—Ag1—P3	112.42 (4)
C22—C27—H27	120.4	P1—Ag1—P2	114.42 (3)
C26—C27—H27	120.4	P3—Ag1—P2	115.70 (3)
C29—C28—C33	118.1 (4)	P1—Ag1—S1	111.13 (3)
C29—C28—P2	118.2 (3)	P3—Ag1—S1	106.10 (4)
C33—C28—P2	123.7 (3)	P2—Ag1—S1	95.37 (4)
C30—C29—C28	121.5 (4)	C10—P1—C4	104.26 (17)
C30—C29—H29	119.3	C10—P1—C16	101.70 (17)
C28—C29—H29	119.3	C4—P1—C16	105.06 (17)
C31—C30—C29	119.6 (4)	C10—P1—Ag1	113.68 (12)
C31—C30—H30	120.2	C4—P1—Ag1	115.71 (13)
C29—C30—H30	120.2	C16—P1—Ag1	114.90 (13)
C30—C31—C32	120.1 (4)	C34—P2—C28	106.06 (17)
C30—C31—H31	119.9	C34—P2—C22	100.12 (17)
C32—C31—H31	119.9	C28—P2—C22	102.89 (18)
C31—C32—C33	120.3 (4)	C34—P2—Ag1	110.73 (12)
C31—C32—H32	119.9	C28—P2—Ag1	116.76 (12)
C33—C32—H32	119.9	C22—P2—Ag1	118.39 (12)
C32—C33—C28	120.4 (4)	C46—P3—C40	102.97 (16)
C32—C33—H33	119.8	C46—P3—C52	103.96 (17)
C28—C33—H33	119.8	C40—P3—C52	101.36 (17)
C35—C34—C39	117.9 (3)	C46—P3—Ag1	117.15 (12)
C35—C34—P2	123.6 (3)	C40—P3—Ag1	114.11 (12)
C39—C34—P2	118.4 (3)	C52—P3—Ag1	115.31 (12)
C36—C35—C34	121.1 (4)		
			
C9—C4—C5—C6	1.2 (6)	C52—C53—C54—C55	−1.2 (6)
P1—C4—C5—C6	−177.9 (3)	C53—C54—C55—C56	1.3 (7)
C4—C5—C6—C7	−1.3 (6)	C54—C55—C56—C57	−0.3 (6)
C5—C6—C7—C8	0.4 (7)	C55—C56—C57—C52	−0.8 (6)
C6—C7—C8—C9	0.4 (6)	C53—C52—C57—C56	0.9 (6)
C5—C4—C9—C8	−0.4 (6)	P3—C52—C57—C56	−176.2 (3)
P1—C4—C9—C8	178.7 (3)	N2—C1—N1—C2	178.2 (4)
C7—C8—C9—C4	−0.5 (6)	S1—C1—N1—C2	−0.3 (6)
C15—C10—C11—C12	−0.1 (6)	N1—C1—N2—C3	−177.9 (3)
P1—C10—C11—C12	−178.0 (3)	S1—C1—N2—C3	0.6 (5)
C10—C11—C12—C13	−0.8 (6)	N2—C1—S1—Ag1	87.7 (3)
C11—C12—C13—C14	1.2 (6)	N1—C1—S1—Ag1	−93.9 (3)
C12—C13—C14—C15	−0.7 (6)	C11—C10—P1—C4	−11.8 (4)
C13—C14—C15—C10	−0.2 (6)	C15—C10—P1—C4	170.3 (3)
C11—C10—C15—C14	0.6 (6)	C11—C10—P1—C16	−120.8 (3)
P1—C10—C15—C14	178.6 (3)	C15—C10—P1—C16	61.3 (3)
C21—C16—C17—C18	−1.1 (6)	C11—C10—P1—Ag1	115.1 (3)
P1—C16—C17—C18	179.9 (3)	C15—C10—P1—Ag1	−62.8 (3)
C16—C17—C18—C19	0.2 (6)	C9—C4—P1—C10	−89.0 (4)
C17—C18—C19—C20	1.3 (6)	C5—C4—P1—C10	90.0 (3)
C18—C19—C20—C21	−2.0 (6)	C9—C4—P1—C16	17.5 (4)
C19—C20—C21—C16	1.2 (6)	C5—C4—P1—C16	−163.4 (3)
C17—C16—C21—C20	0.4 (6)	C9—C4—P1—Ag1	145.4 (3)
P1—C16—C21—C20	179.4 (3)	C5—C4—P1—Ag1	−35.6 (3)
C27—C22—C23—C24	0.8 (6)	C17—C16—P1—C10	36.4 (4)
P2—C22—C23—C24	−177.0 (3)	C21—C16—P1—C10	−142.6 (3)
C22—C23—C24—C25	−1.0 (7)	C17—C16—P1—C4	−72.0 (3)
C23—C24—C25—C26	0.6 (7)	C21—C16—P1—C4	109.0 (3)
C24—C25—C26—C27	−0.1 (7)	C17—C16—P1—Ag1	159.7 (3)
C23—C22—C27—C26	−0.3 (6)	C21—C16—P1—Ag1	−19.3 (3)
P2—C22—C27—C26	177.3 (3)	C35—C34—P2—C28	−74.3 (4)
C25—C26—C27—C22	0.0 (7)	C39—C34—P2—C28	109.2 (3)
C33—C28—C29—C30	0.3 (6)	C35—C34—P2—C22	32.4 (4)
P2—C28—C29—C30	178.2 (3)	C39—C34—P2—C22	−144.1 (3)
C28—C29—C30—C31	0.4 (6)	C35—C34—P2—Ag1	158.1 (3)
C29—C30—C31—C32	−0.8 (7)	C39—C34—P2—Ag1	−18.4 (3)
C30—C31—C32—C33	0.7 (7)	C29—C28—P2—C34	177.1 (3)
C31—C32—C33—C28	−0.1 (7)	C33—C28—P2—C34	−5.1 (4)
C29—C28—C33—C32	−0.4 (6)	C29—C28—P2—C22	72.4 (3)
P2—C28—C33—C32	−178.2 (3)	C33—C28—P2—C22	−109.8 (4)
C39—C34—C35—C36	0.8 (6)	C29—C28—P2—Ag1	−59.0 (3)
P2—C34—C35—C36	−175.7 (3)	C33—C28—P2—Ag1	118.7 (3)
C34—C35—C36—C37	0.2 (6)	C27—C22—P2—C34	−111.7 (4)
C35—C36—C37—C38	−1.1 (6)	C23—C22—P2—C34	65.9 (3)
C36—C37—C38—C39	1.0 (6)	C27—C22—P2—C28	−2.5 (4)
C37—C38—C39—C34	0.0 (6)	C23—C22—P2—C28	175.2 (3)
C35—C34—C39—C38	−0.9 (6)	C27—C22—P2—Ag1	128.0 (3)
P2—C34—C39—C38	175.8 (3)	C23—C22—P2—Ag1	−54.4 (3)
C45—C40—C41—C42	0.5 (5)	C47—C46—P3—C40	−165.2 (3)
P3—C40—C41—C42	−176.7 (3)	C51—C46—P3—C40	12.4 (4)
C40—C41—C42—C43	0.3 (6)	C47—C46—P3—C52	89.4 (3)
C41—C42—C43—C44	−0.9 (6)	C51—C46—P3—C52	−93.0 (3)
C42—C43—C44—C45	0.9 (6)	C47—C46—P3—Ag1	−39.1 (3)
C43—C44—C45—C40	−0.1 (6)	C51—C46—P3—Ag1	138.5 (3)
C41—C40—C45—C44	−0.5 (5)	C41—C40—P3—C46	95.5 (3)
P3—C40—C45—C44	176.6 (3)	C45—C40—P3—C46	−81.6 (3)
C51—C46—C47—C48	0.6 (6)	C41—C40—P3—C52	−157.1 (3)
P3—C46—C47—C48	178.4 (3)	C45—C40—P3—C52	25.8 (3)
C46—C47—C48—C49	−0.6 (6)	C41—C40—P3—Ag1	−32.5 (3)
C47—C48—C49—C50	0.6 (6)	C45—C40—P3—Ag1	150.4 (3)
C48—C49—C50—C51	−0.6 (6)	C57—C52—P3—C46	168.2 (3)
C49—C50—C51—C46	0.7 (6)	C53—C52—P3—C46	−8.8 (4)
C47—C46—C51—C50	−0.7 (6)	C57—C52—P3—C40	61.6 (3)
P3—C46—C51—C50	−178.3 (3)	C53—C52—P3—C40	−115.4 (3)
C57—C52—C53—C54	0.1 (6)	C57—C52—P3—Ag1	−62.2 (3)
P3—C52—C53—C54	177.0 (3)	C53—C52—P3—Ag1	120.8 (3)

### Hydrogen-bond geometry (Å, º)

**Table:**

*D*—H···*A*	*D*—H	H···*A*	*D*···*A*	*D*—H···*A*
N1—H1···O2	0.88	1.89	2.762 (4)	170
N2—H2···O1	0.88	1.91	2.773 (4)	166
