# Supplementary figures and images for: Crystal structure of (1,3-di­methyl­thio­urea-κS)tris­(tri­phenyl­phosphane-κP)silver(I) acetate
Source: Acta Crystallogr Sect E Struct Rep Online. 2014 Aug 30;70(Pt 9):m337–8. doi: 10.1107/S1600536814019047 (PMC4186141; doi:10.1107/S1600536814019047)

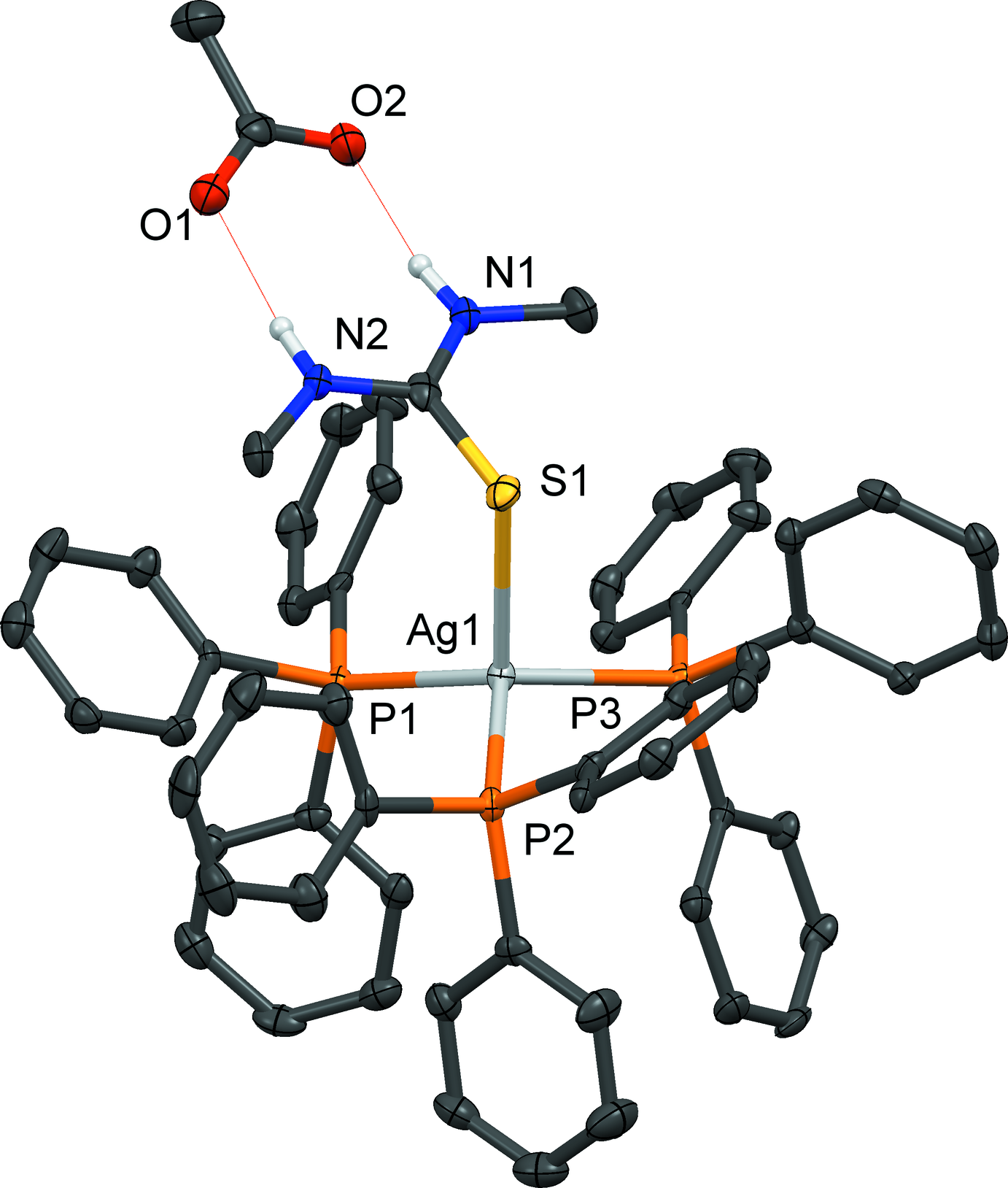

Supplement: Supplementary file 3 [file e-70-0m337-fig1.tif]

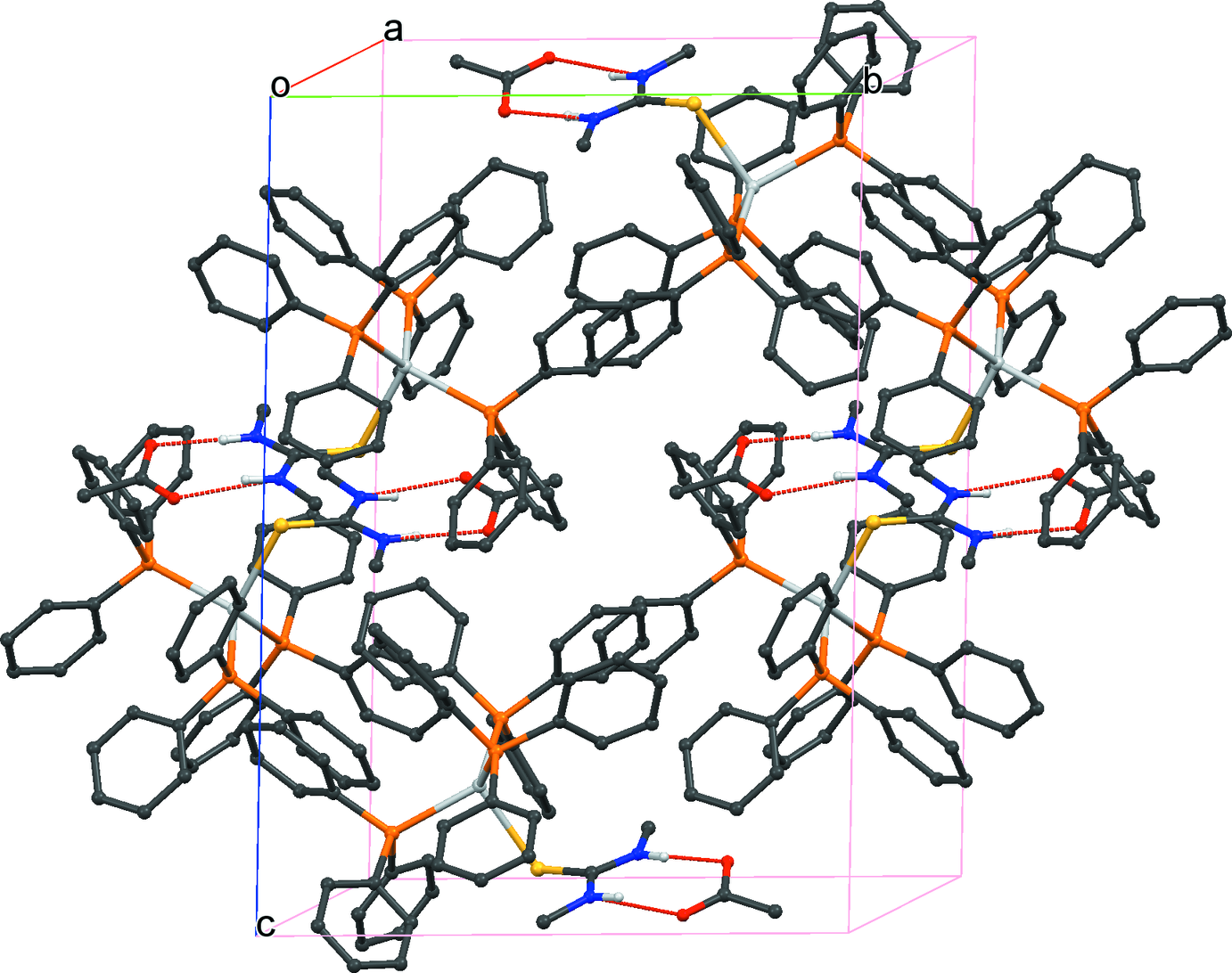

Supplement: Supplementary file 4 [file e-70-0m337-fig2.tif]
